# Human Brown Adipose Tissue and Metabolic Health: Potential for Therapeutic Avenues

**DOI:** 10.3390/cells10113030

**Published:** 2021-11-05

**Authors:** Rajan Singh, Albert Barrios, Golnaz Dirakvand, Shehla Pervin

**Affiliations:** 1 Department of Obstetrics and Gynecology, David Geffen School of Medicine, University of California Los Angeles (UCLA), Los Angeles, CA 90095, USA; shehlapervin@cdrewu.edu; 2 Division of Endocrinology and Metabolism, Charles R Drew University of Medicine and Science, Los Angeles, CA 90059, USA; albertbarrios@cdrewu.edu; 3 Department of Endocrinology, Men’s Health: Aging and Metabolism, Brigham and Women’s Hospital, Boston, MA 02115, USA; 4 Department of Biology, California State University, Dominguez Hills, Los Angeles, CA 90747, USA; gdirakvand1@toromail.csudh.edu

**Keywords:** brown adipose tissue, beige adipose tissue, obesity, cold exposure, uncoupling protein-1, beta-adrenergic receptor, energy expenditure, adipose browning, micro-RNA

## Abstract

Obesity-associated metabolic abnormalities comprise a cluster of conditions including dyslipidemia, insulin resistance, diabetes and cardiovascular diseases that has affected more than 650 million people all over the globe. Obesity results from the accumulation of white adipose tissues mainly due to the chronic imbalance of energy intake and energy expenditure. A variety of approaches to treat or prevent obesity, including lifestyle interventions, surgical weight loss procedures and pharmacological approaches to reduce energy intake and increase energy expenditure have failed to substantially decrease the prevalence of obesity. Brown adipose tissue (BAT), the primary source of thermogenesis in infants and small mammals may represent a promising therapeutic target to treat obesity by promoting energy expenditure through non-shivering thermogenesis mediated by mitochondrial uncoupling protein 1 (UCP1). Since the confirmation of functional BAT in adult humans by several groups, approximately a decade ago, and its association with a favorable metabolic phenotype, intense interest on the significance of BAT in adult human physiology and metabolic health has emerged within the scientific community to explore its therapeutic potential for the treatment of obesity and metabolic diseases. A substantially decreased BAT activity in individuals with obesity indicates a role for BAT in the setting of human obesity. On the other hand, BAT mass and its prevalence correlate with lower body mass index (BMI), decreased age and lower glucose levels, leading to a lower incidence of cardio-metabolic diseases. The increased cold exposure in adult humans with undetectable BAT was associated with decreased body fat mass and increased insulin sensitivity. A deeper understanding of the role of BAT in human metabolic health and its interrelationship with body fat distribution and deciphering proper strategies to increase energy expenditure, by either increasing functional BAT mass or inducing white adipose browning, holds the promise for possible therapeutic avenues for the treatment of obesity and associated metabolic disorders.

## 1. Introduction

The prevalence of obesity and related metabolic complications have significantly increased worldwide. Obesity represents a major global challenge that increases the risk for several chronic diseases, including hypertension, fatty liver disease, diabetes, insulin resistance, dementia, osteoarthritis, sleep apnea and some types of cancer [1]. Thus, obesity presents a substantial burden, both at the individual and population levels, by significantly affecting the quality of life as well as socio-economic productivity. Typical therapeutic approaches targeting dietary intake and physical exercise aimed at reducing obesity and related metabolic complications have not provided long-term health benefits [2,3], suggesting that there remains an unmet need for the development of novel strategies for the treatment and prevention of obesity and associated metabolic complications. Since obesity mainly develops from surplus energy stored in the adipose tissues, therapeutic strategies directed against increasing the energy expenditure and reducing the energy intake, or both, provide attractive avenues to combat obesity-associated metabolic complications. BAT, by virtue of its ability to modulate the organism’s global energy expenditure in the form of heat, has been a subject of tremendous interest to counteract obesity-related diseases. This remarkable capacity of BAT to dissipate energy is mediated via the upregulation of the expression of brown-fat-specific mitochondrial uncoupling protein 1 (UCP1) [4]. In addition to its primary role in regulating thermogenesis, BAT is also involved in cross talk with several peripheral tissues, including the liver, skeletal muscle, gut, the central nervous system and immune cells to control the systemic energy balance and glucose homeostasis [5]. It was widely believed that BAT is present in significant amounts only in newborns, infants and patients with pheochromocytoma, and that it declines with age in adults. However, over the past few years, major technological advancements in clinical studies using ^18^fluoro-2-deoxyglucose positron emission tomography (^18^FDG-PET) scanning in combination with computed tomography (CT) identified functionally active BAT depots in adult humans, which are more frequent in women than in men [6,7,8]. A substantial ^18^F-FDG uptake into the adipose tissue in the supraclavicular and paraspinal regions was observed in adult humans following mild cold exposure compared to the subjects kept at a warm temperature (27 °C). The amount of BAT was found to be inversely correlated with the body-mass index, suggesting the importance of BAT in regulating adult human metabolism [6,7,8].

BAT is also known to secrete various metabolism-improving factors, collectively called BATokines, that target various cell types. Several BATokines, including fibroblast growth factor 21 (FGF21), interleukin 6 (IL-6), growth differentiation factor 15 (GDF15), neuregulin 4 (Nrg4), bone morphogenic protein 8b (Bmp8b), 12,13-diHOME, 9-HEPE and follistatin (Fst), amongst several others, have been reported to serve as BATokines [9,10,11,12,13,14]. Morphological and gene expression data obtained from BAT transplantation studies revealed a larger adipocyte size and a reduced thermogenic gene expression compared to the endogenous BAT, suggesting that the beneficial effects of BAT transplantation could be primarily due to the secreted factors from the transplant [15]. Human pluripotent stem cell-derived brown adipocytes are reported to significantly improve glucose and lipid metabolism and prevent obesity [16,17]. Recent studies demonstrate the association of human BAT with lower blood glucose and triglyceride levels, higher HDL levels, and improved cardio-metabolic health [18]. Future retrospective studies are needed to understand the role of BAT in metabolic health and delineate the clinical and physiological significance of human BAT in the context of obesity-related cardio-metabolic diseases. Besides the well-accepted view that BAT activation is beneficial in general, results of unfavorable outcomes from the hyper-activation of BAT have also been reported in promoting atherosclerosis in animal models [19], cancer cachexia [20] and breast cancer cells in promoting cancer progression [21]. These findings suggest, therefore, that therapeutic interventions for the treatment of obesity-associated metabolic conditions using BAT activation should be carefully controlled and monitored.

## 2. Imaging Studies for Human Brown Adipose Tissue Detection

In spite of early reports of the presence of BAT in adult humans in 1972 [22], its physiological and clinical relevance was mostly ignored by the scientific and medical community. Several simultaneous studies [6,7,8,23] reported the detection of adult human brown adipose tissue, using PET/CT scans, and their confirmation as BAT by molecular analyses of the biopsies from neck and supraclavicular regions in patients undergoing surgery. Considering the need to develop appropriate research protocols to identify depots of metabolic activities following BAT-activating interventions in human subjects, a variety of imaging techniques have been used over the years. Since biopsies of human BAT have been exclusively restricted for ethical reasons and are limited mostly to cadavers or excised tissues obtained for the analysis of oncogenic markers, several biomedical imaging techniques over the years have allowed us to study the functional and morphological characteristics of BAT in vivo.

^18^F-FDG-PET/CT is used as a gold standard for studying activated BAT in humans since 2009, despite its many limitations for identifying and quantifying inducible BAT [24]. The use of ionizing radiation is a major concern when applying PET imaging for BAT, especially in longitudinal studies with healthy human subjects. This technique relies on glucose uptake, but the stored energy in oxidative BAT generated from intracellular triglyceride hydrolysis serves as the main substrate for BAT oxidative metabolism [25]. In addition, evidence for small amounts of glucose uptake by acute stimulation of the β3-adrenergic receptor (β3-AR) has been reported [26]. Therefore, probing oxidative metabolism or fatty acid uptake would be a more sensitive method for molecular imaging of BAT and the assessment of its activity compared to ^18^F-FDG-PET. Nevertheless, from a historical perspective, ^18^F-FDG-PET/CT remains the most widely used imaging study. Studies from the supraclavicular FDG uptake in cancer patients were amongst the first to provide evidence for BAT prevalence in adult humans [27,28]. ^18^F-FDG-PET/CT imaging was capable of detecting higher iBAT levels associated with visceral adiposity. However, the insensitivity of this method to detect inactive BAT in obese and metabolically unhealthy subjects presents a significant limitation of this method in a clinical setting.

Molecular resonance imaging (MRI) and magnetic resonance spectroscopy (MRS) methods do not use radiation and take advantage of the morphological and functional information to differentiate BAT from white adipose tissues (WAT). MRI provides a greater tissue contrast and an adequate spatial resolution compared to other tomographic imaging techniques. The MRI protocol, mostly used to differentiate between WAT and BAT and analyze BAT morphology, is based on the quantitation of water and fat content in the tissue. BAT’s water–fat composition in humans is primarily measured by using chemical shift-encoding water–fat imaging present in the MRI scanners and reported to show a higher reproducibility in humans across all ages [29,30,31,32,33]. However, a proper identification of BAT exclusively based on MRI fat fraction measurements remains challenging, as cold exposure or adrenergic activation significantly change BAT’s lipid content and varies widely with BMI, age, external temperature and diet [22,34,35]. The heterogeneous mixture of BAT and WAT existing in the supraclavicular fossa of human BAT presents another limitation of the water–fat MRI technique. Relatively longer scan times often requiring sedation and use of anesthesia are another practical limitation to MRI. Recently, infrared tomography (IRT) has been used as a valid complementary method to standard ^18^FDG-PET/CT [36]. This method has the advantage of being non-invasive and quick, and could be used in larger studies in human subjects for the detection of BAT. Moreover, near infrared spectroscopy (NIRS) has recently been used for measuring changes in oxygen-dependent light absorption in the tissue in a radiation-free non-invasive manner [37]. Time-resolved NIRS (NIRTRS) could be used to measure the vascular density in the supraclavicular region by estimating hemoglobin (Hb) concentrations, and thereby could provide helpful information on BAT-density [37]. Numerous other ongoing advances in imaging techniques and research protocols using improved tracers with significant improvement in sensitivity and specificity could provide an accurate and reliable assessment of BAT depots as well their metabolic activation. Such advancement in imaging techniques would provide highly improved techniques to measure the mass and activity of human BAT in vivo and understand BAT physiology and its potential as a therapeutic target for the treatment of obesity-associated metabolic complications.

## 3. Sexual Dimorphisms in Rodent and Human Thermogenic Adipose Tissue

The prevalence of obesity and related metabolic diseases is rapidly increasing in both men and women. However, the clinical manifestations of these metabolic diseases significantly differ between the genders. Several retrospective studies over the years have indicated that the prevalence of BAT is higher in women than in men [6,38]. There is also some indication that the metabolic activity and BAT mass is higher in female subjects [38]. However, few studies found no significant difference in BAT activity and mass between men and women following cold exposure [26,39,40,41,42]. In another study, van Marken Lichtenbelt et al. reported that 95% of all young men possessed BAT, implying that it is less likely that women would have a higher prevalence of BAT [7]. The anatomical distribution of BAT is similar in both women and men and mostly in the cervical, supraclavicular, axillary, paraspinal, mediastinal and abdominal areas, of which the supraclavicular regions are the most common location of active BAT detection with ^18^F-FDG-PET/CT [43]. In a diet-induced obesity model, female mice display a higher vascularization of the perigonadal WAT compared to the male mice [44]. It is possible that such gender differences in vascularization could exist in humans, and the sex hormones could differentially mediate the extent of adrenergic activation between men and women. Rodriguez-Cuenca et al. reported that BAT from female rats are more sensitive to β-adrenergic stimulation [45]. Although elevated adrenergic responses to lipolysis are reported in women [46], the underlying differences in β-adrenergic-receptor-signaling between rats and humans could be significantly altered based on the presence of β1-AR in humans [47]. Female rats show an increased abundance of UCP1 expression compared to males and display a higher mitochondrial density [45]. Data from human studies reported that women display a higher cold sensation and may need to activate thermogenesis at higher temperatures compared to men [48]. In addition, female rats show higher protein expression levels of UCP1 and various other proteins involved in thermogenesis compared to male rats fed on a high fat diet [49], supporting the view that rodents display a higher BAT thermogenic capacity in females. In human studies, a similar upregulated UCP1 gene expression was found in the subcutaneous and perirenal WAT of females [50,51].

A systematic global gene expression profile of more than 100 inbred mice shows a higher WAT UCP1 expression and its association with healthier phenotype in female mice [52]. On the other hand, UCP1 expression positively correlated with increased fat mass and insulin resistance in male mice. Collectively, these reports support sexual differences in BAT prevalence and function.

## 4. Aging-Induced Changes in Beige and Brown Adipocytes

Adipose tissue undergoes profound changes with aging in terms of its composition and distribution, leading to metabolic alterations. This aging-related adipose tissue dysfunction has a severe impact on whole-body energy homeostasis and often results in the progressive development of metabolic complications. It has been established that human BAT mass and activity decline with age. BAT formation starts during gestation, where it is critical for thermogenesis in early phases of human life. Supraclavicular BAT is the major site of metabolically active BAT in childhood. BAT activity increases during adolescence and sexual maturation and declines with aging. Cold-stimulated BAT activity by ^18^F-FDG-PET/CT imaging is rarely detectable in living individuals over the age of sixty, although the BAT mass may not change [53]. A significant aging-related decline of BAT activity and a concomitant loss of UCP1 expression in rodents have also been reported in several studies, suggesting that a gradual decline of BAT activity is common in both rodents and humans [54].

Possible mechanisms involved during the aging-related impairment of BAT development and function include loss of mitochondrial function, reduced sensitivity to sympathetic tone, alterations in brown adipose progenitor/stem cell function and changes in the endocrine control of BAT formation [55,56]. A reduced capacity of beige adipocyte formation, resulting from aging-induced changes in trophic factors in the adipose tissue, also contributes to the impairment of the thermogenic function [57]. Mitochondrial dysfunction has long been associated with several aging-related disorders, including obesity, type-2 diabetes, tumorigenesis and neuronal diseases [58]. Increased mitochondrial DNA mutations and a progressive decline in mitochondrial biogenesis are important contributors to human aging-associated adipose tissue dysfunction [59]. The age-associated impairment of the regenerative potential of brown adipose stem/progenitor cells could contribute to dysfunctional BAT adipocytes [58]. The defective differentiation ability of the CD137- and TMEM26-expressing sub-population of WAT progenitor cells could also contribute to the loss of beige adipocyte during aging [60]. SirT1, a potent inducer of adipose browning, can significantly enhance the beige adipocyte differentiation capability in an elderly adipose-derived mesenchymal stem cell (AD-MSC) population [61]. The sympathetic nervous system (SNS) plays a central role in the recruitment of brown adipocytes and its thermogenic activity through β3-AR stimulation [62]. SNS stimulation and BAT activation were lower in older lean men compared to the younger and obese men [63]. In addition, Yoneshiro et al. reported that a single nucleotide polymorphism in β3-AR and UCP1 contributed to an aging-associated decline in BAT activity in humans [64], suggesting that the aging-related decrease in SNS stimulation and sensitivity in older human may result in a decreased ability to recruit and activate BAT.

**Figure 1:**
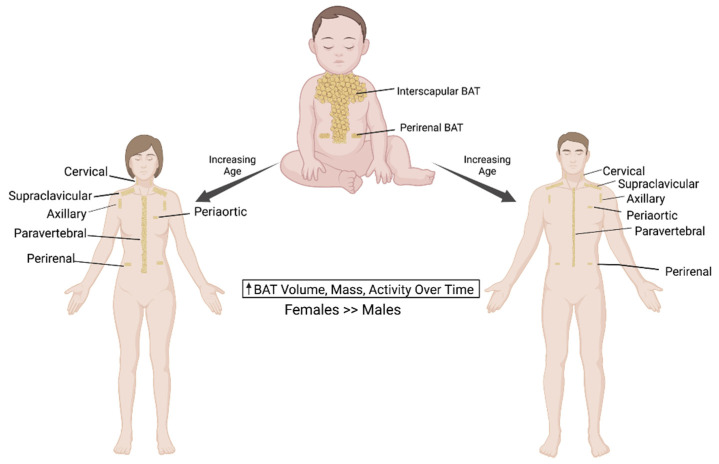
BAT distribution in infants, women and men. BAT is stored in a separate interscapular depot in infants, and they lose their brown adipose tissue as they age. In adult humans, most brown adipocytes can be found in the supraclavicular BAT depots in the neck region. Lesser amounts of BAT are found across the aorta, vertebrae, axillary and kidney areas. There is a similar distribution of brown adipose tissue in both women and men. However, women have a greater amount of BAT mass and activity.

Aging-associated hormonal changes also play a central role in regulating the thermogenic activity in human BAT. While sex hormones like estrogens and androgens positively regulate BAT activity and function [65,66], glucocorticoids such as dexamethasone reduce the catecholamine-induced expression of UCP1 [67]. Relatively higher levels of sex hormones in the young counteract the generally inhibitory effects of glucocorticoids in BAT. Because of aging, sex hormone levels significantly decline without a significant alteration in glucocorticoid levels, resulting in reduced energy expenditure and the loss of BAT activity. The aging-associated decrease in thyroid hormone (TH), known regulators of white adipose browning, has recently been demonstrated in murine WAT [68,69]. In light of these reports, future human studies are needed to develop effective pharmacological interventions aimed at preventing the aging-associated decline in BAT activity. Figure 1 summarizes the BAT distribution in infants, women and men.

## 5. Molecular Gene Signature of Brown and Beige Adipocytes in Rodents and Humans

Various cell-sorting and lineage-tracing studies in rodents have demonstrated that different types of adipose cells have distinct sets of molecular markers and arise from distinct pools of progenitors [60,70,71,72]. Classical brown adipocytes present in BAT originate from a population of dermomyotomes expressing Myf5, Pax7 and engrailed 1 [73,74]. Analyses of the global gene expression profile of interscapular BAT demonstrate that these brown adipocyte precursors resemble the gene signature, with skeletal muscle cells that also express a Myf5-positive precursor population [75]. Data from the proteomics analysis of interscapular BAT and skeletal muscle further confirmed a strong similarity in the protein levels [76]. Classical BAT is reported to selectively express high levels of *Lhx8*, *Zic1*, *Eva1* (*Mpzl2*) and *Epsti 1*, although there are recent reports that suggest that *Lhx8* could also be present in human beige adipocyte [77]. Several additional markers, including *Ebf2*, *Pdk4*, *Hsbp7*, *Oplah*, *Fbxo3*, *Slc29a1* and *Acot2*, were found to be highly enriched in interscapular BAT obtained from 129SVE mice [60]. Recently, *Prex1*, a positive regulator of *Ucp1* gene expression, was found to be another BAT marker present in the brown adipose precursor cells [78]. Since no published studies have reported the upregulation of *Prex1* levels in beige adipocytes, it could be considered as a unique BAT marker.

Beige adipocytes, on the other hand, arise from a non-Myf5 lineage [79], although there are several recent reports that beige adipocytes in WAT depots may have multiple origins [50,72]. Beige adipocytes, in general, are selectively enriched in *Tmem26*, *CD137*, *CD40* and *Tbx1*. These markers are expressed in high levels in epididymal adipose depots compared to the interscapular BAT following cold exposure or β-adrenergic stimulation [60]. Several additional beige-selective genes, including *Klh113*, *Ear2*, *Sp100*, and *Slc27a*, were reported in the same study following the analysis of gene expression in inguinal WAT and BAT. Gene expression and a histological analysis of human BAT that resemble beige adipocytes identified *Cited1*, *Hoxc8*, *Hoxc9* and *Shox2* as beige-selective genes [77,80]. Differences in the levels of microRNA (miRNA) between beige and BAT have also been identified. The Mir193b-365 cluster was found to be a key regulator of BAT development [81]. Additional miRNAs required for the maintenance and differentiation of BAT, including miRNA182 and miRNA 203, were identified following a comparative miRNA expression profiling of mouse and human BAT [82,83]. Some recently identified miRNA, including miRNA196b and miRNA26, are reported to positively and negatively regulate the brown adipogenesis of white fat progenitor cells, respectively [84,85].

Transcriptional analyses of human BAT isolated from multiple adipose depots display molecular signatures that closely resemble beige adipose-selective genes. The authors found that common beige adipose markers including *HOXC8*, *HOXC9*, *CITED1*, *CD137*, *TMEM26* and *TBX1* were highly expressed in all human BAT [77]. A molecular characterization of human perirenal BAT depot shows a higher expression of *HMGCS2*, *CKMT1A/1B*, *KCNK3* and *PGC1-α* genes in BAT compared to the WAT [86]. They also identified high levels of BAT-associated genes, including *ACOT11*, *PYGM* and *FABP3*, in the perirenal adipose tissue, which display a high correlation with UCP1 and to each other. Recent studies in humans indicate high levels of UCP1-immunopositive adipocytes in the anatomically defined neck fat from the supraclavicular and spinal cord regions of adult human subjects and display molecular signatures of classical BAT, including high expression levels of genes involved in mitochondrial biogenesis and thermogenesis, including *UCP1*, *PGC1α* and *DIO2* [87]. The degree of correlation of the genes assessed by hierarchical clustering in deep neck tissues with the highest UCP1 expression show increased levels of *UCP1*, *ZIC1* and *LHX8*. Some genes previously designated as markers of BAT lineage in mice, including *EBF3*, *MPZL2* and *FBXO31*, did not have an elevated expression in the deeper human neck depot or associate closely with *UCP1* expression. On the other hand, the expression levels of *CD137*, *TMEM26*, *TBX1*, *EVA1* and *EBF3* does not differ between subcutaneous and deeper neck adipose depots. Since adipose tissue depots are highly heterogeneous and inter-subject differences could significantly affect the thermogenic potential, clonal cell lines from human neck fat were generated and characterized for their adipogenic and metabolic functions in vitro and in vivo after transplantation into immune-deficient nude mice [78]. Using the UCP1 reporter system, the authors were able to reveal two general categories of genes in preadipocytes with enhanced capability to regulate the late-stage thermogenic differentiation program. *PREX1*, *CTTNBP2*, *ACTC1* and *SSTR1* genes were identified in the first category as positive regulators with enhanced thermogenic capacity. On the other hand, in the second category, *DMRTA1*, *EDNRB*, *FAT1* and *PTPRB* were reported to have a negative correlation with UCP1-luciferase levels [78]. Zuriaga et al., performed comparative gene analyses of browning gene expression profiles in the subcutaneous and visceral WAT of obese mice and human subjects and reported a pattern that is opposite to what is observed in mice [88]. Obese mice displayed a higher expression of typical browning markers *UCP1*, *Cidea* and *Prdm16* and beige markers *Tbx1* and *P2rx5* compared to the epididymal WAT. However, in obese humans, visceral WAT show a higher expression of UCP1 and several other adipose browning (*CIDEA*, *PRDM16*, *TBX1* and *P2RX5*) and mitochondrial (*COX8B*, *PGC1α*, *ATP5A* and *NDUFA1*) genes compared to the subcutaneous adipose depots [88]. These findings suggest an increased browning capacity of human visceral WAT compared to the subcutaneous WAT.

## 6. Cold Exposure and Activation of Sympathetic Nervous System

In earlier experiments, the appearance of brown-like adipocytes in the parametrial fat depots was reported in mice following severe cold exposure [89]. Subsequent studies in murine models over the years clearly established a role for cold-induced BAT activation as a physiological response to acute or mild cold exposure for maintaining thermal demands through non-shivering thermogenesis (NST) [90,91]. Cold acclimation led to the activation of PGC1α and the induction of a UCP1-PRDM16-signaling cascade in murine studies [92].

The higher prevalence of BAT during cold season and the reported seasonal variability in human subcutaneous and retroperitoneal WAT strongly suggest that cold exposure should promote adipose browning and the activation of BAT characteristics in humans [92,93]. Cold exposure is the most common method for BAT activation in humans, via the activation of NST [94], that is achieved either by reducing the ambient room temperature between 16 and 19 °C [95] or with cold water-infused jackets [96,97]. Cold acclimation increases the BAT’s oxidative capacity, which correlates with a reduction of shivering thermogenesis [94,98]. Cold adaptation in BAT is also associated with mitochondrial remodeling and vascularization for adaptive thermogenesis [91] and fatty acid oxidation through UCP1 during high metabolic demands [99].

A significant increase in BAT activity and NST was reported in humans following 10-day acclimation protocols [94]. Respiratory measurements in skeletal muscle suggested no significant contribution from mitochondrial uncoupling towards increased NST. The authors did not find any sex differences in BAT prevalence and activities between men and women. There was no significant increase in adipose browning markers in subcutaneous WAT, suggesting that a stronger cold stimulation or prolonged period of cold acclimation may be needed for the upregulation of general browning markers. In another human study, Ouellet et al. reported that BAT’s oxidative metabolism significantly contributed to an increased energy expenditure, glucose uptake and non-esterified fatty acid (NEFA) turnover in healthy men during acute cold exposure [96]. A similar increase in glucose uptake following the activation of BAT by cold exposure and insulin was reported in another study [100], suggesting that BAT activation leads to the clearance of plasma glucose. The analysis of glucose metabolism in individuals with (BAT+) or without (BAT−) BAT under cold exposure showed an increased whole-body glucose in the fasted and insulin- stimulated BAT+ individuals. This only suggests that BAT can indeed significantly influence systemic glucose metabolism in humans [101] and may represent a novel target to combat hyperglycemia. In addition, it was observed that a mild daily cold exposure (17 °C) for 6 weeks induces BAT recruitment in healthy human subjects with low BAT activity independently of age and fat-free mass [42], suggesting that the cold-induced recruitment of BAT could serve as a viable means to decrease fat mass in aging populations.

The sympathetic nervous system plays an important role in the physiological regulation of BAT activity, mediated mainly via the activation of the beta 3-adrenergic receptor (β3-AR) present in brown adipocytes. The chronic treatment of rats with selective β3-AR agonist CL316243 significantly increased the energy expenditure and body temperature, and these effects were associated with a 3–4 fold increase in *Ucp1* expression in interscapular BAT [42,102]. In this study, CL316243 not only promoted BAT’s mitochondrial biogenesis, but it also inhibited the development of WAT hyperplasia during diet-induced obesity. The treatment of male mice with CL316243 led to a significant upregulation of the *Ucp1* gene expression in retroperitoneal adipose tissue and correlated with weight loss [102,103]. These studies conducted in rodents support the view that the activation of β3-AR with CL316243 is an effective method to induce BAT activity and stimulate the beige cell phenotype in typical WAT. The long-term sympathetic regulation of thermogenic BAT activity and its association with reduced body weight in catecholamine-producing human pheochromocytoma patients is well known. The administration of β3-AR agonist in human subjects for 4–8 weeks significantly improved insulin sensitivity and lipid profiles without any change in body weight [104,105]. More recently, the treatment of human subjects with a single high dose of β3-AR agonist mirabegron increased BAT’s ^18^F-FDG uptake, and the energy expenditure [106], although β2-AR and β1-AR are more highly expressed in human BAT [107]. Mixed AR agonist ephedrine also increased the ^18^F-FDG uptake in BAT in lean but not obese subjects at room temperature [108]. However, the side-effects associated with increased heart rate and blood pressure induced by these compounds significantly limit the potential use of this therapeutic approach [106,108,109].

## 7. WAT Browning Factors

Due to the relatively lower amount of BAT present in adult humans, WAT has remained the major focus of targeting against obesity and related metabolic diseases. Even though WAT is known to have fewer numbers of mitochondria with larger lipid droplets, the manipulation of WAT to brown-like phenotype with increased thermogenic capacity through the process called “adipose browning” is being explored as a major therapeutic strategy. Several browning agents have been identified in recent years in animal studies, some of which are currently being investigated in humans [110,111]. The safe activation of adipose browning requires the identification of the specific molecular pathways involved in WAT browning. Fibroblast growth factor 21 (FGF21), expressed mainly in the liver [112], is also present in BAT [113] and regulates energy metabolism by promoting adipose browning and lipoprotein catabolism [114,115]. An increased FGF21 expression in adipose tissues was observed within 6 h of cold exposure without any change in its circulating levels [114]. Using differentiated brown and inguinal adipocytes derived from PGC-1α-knockout (KO) and PGC-1α Flox mice, Fisher et al. demonstrated that PGC-1α is required for FGF21-induced thermogenesis in adipose tissues [114]. The induction of PGC-1α by FGF21 was mostly regulated at the post-translational level. This induction of the PGC-1α protein content in brown and WAT was significantly attenuated in FGF21-KO mice, suggesting an important role for PGC-1α during FGF21-induced adipose browning [114]. The therapeutic potential of FGF21 analogs has recently been explored in obese human subjects with type-2 diabetes [116,117]. These analogs reduced body weight and improved circulating lipids but failed to improve glucose levels. These studies did not measure BAT activity or WAT browning. Additional endocrine factors including natriuretic peptides, prostaglandins and β-aminoisobutyric acid (BAIBA) and thyroid hormones (TH) are known to influence WAT differentiation and browning [118,119,120]. Several members of the transforming growth factor beta (TGF-β) superfamily, including bone morphogenic proteins (BMP) such as BMP7, BMP8b and BMP4, regulate various aspects of WAT browning and BAT differentiation in rodents, as well as in human adipocyte cell models [121,122,123]. Additional modulators of the TGF-β signaling pathway, including follistatin (Fst) and inhibitors of myostatin (Mst), have been recently reported to upregulate key thermogenic markers, increase mitochondrial biogenesis and promote WAT browning [124,125,126]. Improved insulin sensitivity and reduced lipogenic effects of Gleevec were associated with increased WAT browning and energy expenditure. These beneficial effects of Gleevec were blocked by the inhibition of PPARγ phosphorylation, suggesting a critical role for the PPARγ-mediated signaling pathway during the process [127]. Thiazolidinediones (TZDs), known agonists of PPARγ used as insulin sensitizing drugs for the treatment of T2D, promote adipose browning both in vivo and in vitro [128,129]. Another antidiabetic agent, liraglutide, that acts as an agonist of the glucagon-like peptide-1 (GLP-1) receptor, significantly reduced the weight gain in obese subjects [130]. Although the GLP-1 mediated weight loss results were mainly due to appetite suppression, increased energy expenditure is recently reported in liraglutide-treated obese human patients with type-2 diabetes [131]. The bile acid chenodeoxycholic acid (CDCA) increases BAT activity in both mice and humans [132,133]. Several plant-derived products influence adipocytes’ metabolic characteristics. Natural plant polyphenols such as resveratrol and butein increase the energy expenditure and promote thermogenesis by the induction of *Prdm16*, *Ucp1* and *Pgc-1α* [134]. Another plant-based compound, capsaicin, stimulates the thermogenic *Ucp1* and *Bmp8b* genes and triggers WAT browning by stimulating the SirT1-dependent acetylation of PPARγ and Prdm16 [135]. Another study in humans using a single dose of capsinoids demonstrated a selectively increased energy expenditure in people with metabolically active BAT, suggesting the beneficial effects of capsinoid-induced WAT browning in both humans and mice [136]. Other polyphenols, including tea catechin, have also been suggested as safe and effective therapeutic options to promote white adipose browning [137]. Lactate, an important metabolic intermediate, upregulates *Ucp1* expression and induces WAT browning by regulating PPARγ signaling [138].

## 8. Exercise

Exercise is an effective way to prevent and treat obesity and related metabolic diseases. Although the adaptation to skeletal muscle is most widely studied, it also significantly influences the phenotype and functions of adipose tissues. Exercise increases whole-body energy expenditure and several metabolic adaptations to WAT through the process of adipose beiging mainly in subcutaneous WAT (scWAT). Exercise induces the upregulation of thermogenic genes such as *Prdm16* and *Ucp1* in scWAT and increases the presence of multilocular lipid droplets in adipocytes [139,140]. PGC1-α, a muscle-derived myokine, seems to be an important mediator of the exercise-induced browning process that influences myogenesis as well as mitochondrial oxidative phosphorylation [141]. Irisin, another browning agent that works through the PGC1-α pathway, is secreted from skeletal muscle and upregulates UCP1 expression in scWAT, increases energy expenditure and protects against diet-induced weight gain in mice [141]. The irisin-induced adipose browning of white adipocytes in vitro and its circulating levels are increased in humans following exercise and cold exposure [141,142]. However, human data obtained from several laboratories studying the effect of exercise on irisin levels are inconsistent and raise doubts about the role of irisin in mediating exercise effects [143,144]. Although the direct effects of irisin administration in humans have not yet been conducted, in some human studies plasma irisin levels were found to be higher in obese humans and its levels correlate positively with plasma FFA [145,146]. In light of these controversial reports, the beneficial role of irisin for treating or managing obesity has been questioned. Several other exercise-induced factors, including FGF21, IL-6, follistatin, myostatin, meteorin-like1 (Metrnl), lactate, BAIBA and brain-derived neurotrophic factor (BDNF) have been associated with WAT browning [119,147,148,149,150]. Exercise-induced changes in WAT include increased mitochondrial biogenesis and glucose uptake, decreased lipid content and inflammation [151,152]. Although such adaptations to exercise in multiple tissues are known in both rodents and humans, it is important to note that more investigations are needed to clearly establish exercise-induced adipose browning and whole-body metabolic health in human subjects.

## 9. MicroRNAs in Browning

Micro RNAs (miRNAs) are single stranded small non-coding RNAs (20–24 nucleotides) that regulate a wide spectrum of biological processes implicated in cellular metabolism and the pathology of a variety of diseases. In recent years, several miRNAs were identified that play important roles in regulating beige and brown adipocyte differentiation and function [153,154]. Delineating the regulatory mechanisms of miRNA action has great potential to provide therapeutic approaches to combat obesity and related metabolic diseases by increasing the overall energy expenditure. Several miRNAs, including miRNA328, miRNA193b-365, miRNA203, miRNA182, miRNA129, miRNA106b and miRNA93b, have been identified that specifically regulate brown adipogenesis either by promoting or inhibiting the BAT thermogenic program [153]. The inhibition of miRNA182 or miRNA203 results in a significant reduction of various BAT markers, including *UCP1*, *PGC1-α* and *Cidea*, as well as the downregulation of genes involved in the electron transport chain and oxidative phosphorylation [83]. However, there was no change in the levels of the classical adipogenic markers *PPARγ* and *FABP4* in this study. Interestingly, the platelet-derived growth factor receptor alpha (Pdgfrα) and insulin-induced gene 1 (Insig1) were identified as targets of miRNA182 or miRNA203 [83]. The miRNA193b-365 cluster is highly expressed in BAT and is reported to promote brown fat differentiation by inhibiting the runt-related transcription factor 1 translocated to 1 (Runx1t1) [81]. The function of these miRNAs is still not confirmed, as in-vivo studies show no change in BAT morphology and function in the absence of this cluster. The levels of miRNA328 in mice on a high-fat diet were decreased in BAT and found to be upregulated during brown adipocyte differentiation [155]. On the other hand, a decreased expression of several thermogenic genes, including *Ucp1*, *Prdm16*, *Pgc-1α* and *Cidea*, was reported after the inhibition of miRNA328 in this study. In addition, the overexpression of miRNA328 in brown adipocytes significantly inhibited the Bace 1 protein, that decreases energy expenditure and UCP1 expression [156]. The MiRNA106b-93 cluster is identified, as negative regulators of BAT differentiation and their levels are found to be increased in obese mouse BAT [157]. The inhibition of miRNA 106-93 induced the expression of several BAT markers, including UCP1, PRDM16, PGC1-α and Cidea [157]. These findings suggest, therefore, an important role for the miRNA106b-93 cluster in BAT energy homeostasis. The circulating levels of liver-specific miRNA122 were increased in obese individuals and found to be negatively associated with BAT activity [158].

In addition, several miRNAs play a significant role in regulating beige adipocyte development and function. A better understanding of the key beige-specific miRNAs and their regulation would be critical for developing efficient therapeutic approaches to increase cellular energy expenditure and slow down the progression of obesity. miRNA196a plays an important role in the browning of white adipose progenitor cells, with increased expression of several brown adipogenic genes, such as *C/ebpα*, *Prdm16*, *Ucp1* and *Pgc1-α* [84]. MiRNA196a induces WAT beiging in humans by the direct binding and suppression of homeobox C8 (Hoxc8), a known repressor of C/EBPβ. The overexpression of miRNA196a improved glucose metabolism and resistance to diet-induced obesity in mice [84]. The MiRNA26 family (miRNA26a and miRNA26b) has been identified as an important regulator of white and beige adipocyte differentiation in humans [159]. The MiRNA26 level is induced in WAT following cold exposure and mimics of miRNA26a/b promote adipose browning and the mitochondrial phenotype by upregulating the expression of UCP1, PGC1-α and aP2. Mechanistically, the adipose browning effects of the miRNA26 family are mediated by the direct targeting of ADAM17 [159]. MiRNA125-5p has been identified as a negative regulator of adipose browning. Its expression level is higher in WAT compared to BAT and decreases during browning [160]. Beige adipocyte formation and mitochondrial biogenesis is significantly inhibited following the injection of miRNA125-5p into WAT [161].

In addition, several miRNAs have been identified that play important roles in regulating both BAT and WAT browning. MiRNA30 family members promote the development of beige and thermogenesis [162]. The levels of miRNA30 are higher in BAT compared to WAT. The activation of β-AR signaling or cold exposure upregulates the expression of key thermogenic (*UCP1* and *Cidea*) and mitochondrial biogenesis genes in brown adipocytes. The inhibition of miRNA30b/c levels in vivo results in the downregulation of UCP1 expression and the reduction of BAT mitochondrial respiration by its direct action on the targeting of receptor-interacting protein 140 (RIP140) [163], suggesting that miRNA30b/c positively regulates beige adipocyte development as well as BAT thermogenesis. MiRNA455 was identified as a key regulator of brown adipogenesis in multipotent mesenchymal cells. MiRNA455 levels are induced by cold exposure and BMP7 treatment [160]. The inhibition of miRNA455 results in the suppression of brown adipose differentiation. In addition, adipose-specific transgenic mice overexpressing miRNA455 display significant WAT browning, increased insulin sensitivity and glucose tolerance and resistance to a diet-induced increase in body weight [160]. MiRNA378 has been identified as the only miRNA that can regulate brown and white adipocytes in opposing fashion. The adipose-specific overexpression of miRNA378 increased the BAT mass and brown adipogenesis but showed a decreased WAT mass and cell size, and reduced diet-induced obesity in ob/ob mice [164]. These dual effects of miRNA378 in promoting brown adipogenesis but blunting WAT beige adipogenesis are caused by the direct targeting of Pde1b, a phosphodiesterase that causes a reduction in cAMP levels [165]. Table 1 briefly summarizes various miRNAs involved in brown/beige fat development and their targets [83,84,85,155,156,157,158,159,160,161,162,163,164,165,166,167,168,169]. Further studies are needed to clearly understand the complex mechanism in order to develop suitable miRNA-based therapeutic approaches for the treatment of obesity and related metabolic disorders.

## 10. Challenges and Potential Concerns with BAT Activation as a Therapeutic Strategy to Combat Obesity

The therapeutic targeting of BAT for obesity was envisioned in late 1970s based on rodent studies, suggesting that BAT may be the major site for thermogenesis in response to both cold and diet [170,171]. The unequivocal discovery of active and functional BAT in healthy human subjects confirmed the expression of thermogenic UCP1 and its energy-dissipating capacity in humans. Increasing energy expenditure by cold exposure and BAT activation holds great potential to combat obesity-related metabolic complications; however, there are notable concerns with this approach that must be carefully evaluated. While there are many similarities between mouse and human BAT, notable morphological and anatomical differences also exist. In order to translate the significance of rodent studies in humans, it is important to generate proof of concept studies in humans for successful clinical outcomes. The activation of the β-AR signaling needed for BAT thermogenesis is mediated via β3-AR in rodents. Recent studies in humans revealed that β2-AR is the predominant adrenergic receptor [107], suggesting that a selective BAT activation in humans may be difficult to achieve and requires the activation of another β-AR subtype. Targeting thermogenic BAT activation could result in a significantly increased heart rate, as the increased energy expenditure requires an increased demand for oxygen. A chronic BAT activation from β3-AR agonists or thyroid hormone replacement may cause tachycardia and hypertension, resulting in unwanted cardiovascular side effects such as stroke or myocardial infarction [172,173]. Another potential problem during the pharmacological activation of BAT could result from the dissipation of heat. Weight loss medication 2,4-dinitrophenol (DNP), that works through the uncoupling of oxidative phosphorylation, causes hyperthermia and could be fatal at higher doses [174]. Although it is unlikely that selective pharmacological approaches to activate thermogenesis will have severe thermal stress at low doses, caution is warranted to avoid unwanted off-target effects. In addition, the impact of increased energy expenditure could lead to a compensatory hyperphagic response during cold exposure [175]. However, data accumulated over the past decade indicate that pharmacotherapy approaches to activate BAT via β3-AR agonists or the administration of various thermogenic food ingredients lead to increased food intake [176].

**Figure 2:**
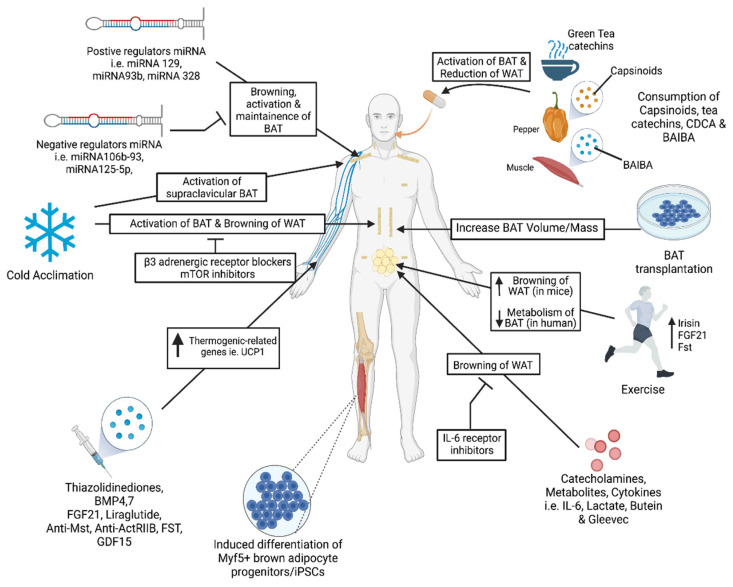
Therapeutic interventions to increase BAT mass and activity. Acute or chronic cold exposure activates BAT metabolism or induces BAT browning. β3-AR blockers and mTOR inhibitors inhibit the browning of WAT and the activation of BAT. Exercise activates BAT and induces WAT browning via the upregulation of irisin, follistatin (Fst) and FGF21 (fibroblast growth factor 21). Intravenous (IV) administration of proteins, i.e., FGF21, growth and differentiation factor 15 (GDF15), bone morphogenic protein 7 (BMP7) and follistatin (FST), or anti-Activin receptor type 2B (ActRIIB), anti-Myostatin (Mst) antibodies and various pharmaceutical agents, including Liraglutide and thiazolidinediones, upregulate thermogenic genes like *UCP1* to induce the browning of WAT. WAT browning is also induced through the use of Gleevec, lactate and Butein. Certain cytokines, catecholamines and metabolites were used for the browning of WAT. IL-6 receptor inhibitors block the browning of WAT. BAT transplantation increases BAT mass and activity. MiRNAs target regulatory mechanisms that regulate thermogenesis and the browning of WAT either positively or negatively. Trans-differentiation of induced pluripotent stem cells and mesenchymal progenitor cells into brown adipocytes by targeting regulatory mechanisms to overexpress thermogenic and adipogenic genes. Diet or consumption of oral supplements known to activate BAT, like capsinoids, tea catechin and BAIBA can be extracted and utilized as an IV injection or as an oral supplement.

Several recent studies also suggest that BAT activation could be harmful. A cold-induced BAT hyper activation in ApoE-null mice resulted in the exacerbation of atherosclerotic plaques and their stability [19]. This cold-induced lipolysis in ApoE-null mice was food-intake-independent and significantly increased the LDL remnants. Increased levels of inflammatory cells and plaque-associated micro vessels were detected in cold-acclimated mice. The deletion of UCP1 in BAT protected these ApoE-null mice from the development of cold-induced atherosclerotic lesions. Adverse effects of BAT activation are also reported in the development of cancer cachexia [177]. Interleukin-6 (IL6) and growth and differentiation factor 15 (GDF15), two BAT-secreted factors, are key mediators of cancer-induced cachexia [177,178]. Using xenotransplantation models that use human breast cancer cells as well as fresh human tumor tissues transplanted into nude mice, Singh et al. provided evidence for the involvement of beige and brown adipose cells during the progression of breast cancer [21]. In this study, a selective enrichment of BAT-selective genes was observed in the tumor as well as the host cells, and a depletion of UCP1 and the Myf5-positive population resulted in a significantly reduced tumor size. It is not known whether BAT plays an important role in the progression of other cancers. These findings clearly indicate, therefore, that BAT-based therapeutic drug development for the treatment of obesity and related diseases should be carefully assessed to avoid unwanted adverse effects. Figure 2 summarizes a brief overview of several therapeutic interventions to increase BAT mass and activity.

## 11. Conclusions

Since the demonstration of active and functional BAT in adult human subjects, the activation of adipose browning characteristics to dissipate excess stored energy has shown great promise to combat obesity-associated comorbidities including type-2 diabetes, insulin resistance, hyperlipidemia and cardiovascular diseases. The identification of novel compounds that can promote adipose browning and understand the molecular signaling mechanism involved during the process will provide new insights for therapeutic drug development targeting obesity-related diseases. Recent studies have also provided ample evidence that BAT also plays an important role in the regulation of systemic and peripheral glucose homeostasis by functioning as a metabolic sink for glucose and triglycerides. A number of pharmacological agents have been tested to activate the adipose browning characteristics (Table 2). However, these attempts have mostly failed, partially due to their modest efficiency and adverse side effects, including elevated blood pressure [108]. It is also important to develop new techniques to assess the thermogenic capacity of novel compounds in adult humans, as ^18^F-FDG-PET/CT scans do not accurately measure the total BAT mass. Other currently available devices are not sensitive enough to accurately detect UCP1-expressing subcutaneous WAT and other adjacent tissues. Recent studies demonstrating the therapeutic potential of transplanted BAT in rodent studies to improve weight loss and improved insulin sensitivity have not been explored in human subjects. The effectiveness and clinical relevance of BAT activation and recruitment in human subjects needs to be further explored in detail.

## Figures and Tables

**Table 1:** MicroRNAs involved in brown/beige fat development and their targets.

miRNA	Role in Brown/Beige Fat Development	Target(s)	Species/Reference
miRNA328	Positive regulation of BAT differentiation	*Bace1*	Mouse [155,156]
miRNA 193b-365	Regulation of BAT differentiation	*Runx1t1*	Mouse [81]
miRNA203	Promotes BAT adipogenesis	*Insig 1*, *Pdgfr 2*	Mouse [83]
miRNA182	Promotes BAT adipogenesis	*Insig 1*, *Pdgfr 2*	Mouse [83]
miRNA129	Positive regulator of BAT function	*Igf2*, *Egr1*	Human, Mouse [166]
miRNA106b	Negative regulator of BAT differentiation	*Ppara*	Mouse [157]
miRNA34a	Negative regulator of BAT and beige adipogenesis	*Fgfr1*	Mouse [167]
miRNA27b	Negative regulator of BAT and beige adipogenesis	*Prdm16*, *Ppara*, *Pparg*, *Creb*	Mouse [168]
miRNA93b	Negative regulator of BAT differentiation	*Ppara*	Mouse [157]
miRNA196a	Promotes adipose browning	*Hoxc8*	Mouse [84]
miRNA26a/b	Promotes white and beige adipocyte differentiation	*ADAM17*, *Fbx19*	Human, Mouse [159]
miRNA125-5p	Negative regulator of adipose browning and mitochondrial biogenesis	*MMP11*	Human [161]
miRNA30b/c	Positive regulator of BAT and beige adipocyte development	*Rip140*	Mouse [162,163]
miRNA455	Positive regulator of BAT and beige adipose browning	*Runx1t1*, *Necdin*, *Hif1an*	Human, Mouse [160]
miRNA378	Promotes BAT mass and brown adipogenesis but negatively regulates beige adipogenesis	*Pde1b*	Mouse [164,165]
miRNA122	Negatively regulates BAT activity	*CD320*, *AldoA*, *BCKDK*	Human [158]
miRNA133	Negative regulator of BAT differentiation	*Prdm16*	Mouse [85]
miRNA155	Negative regulator of BAT differentiation	*C/ebpβ*	Mouse, Human [169]

**Table 2:** Adipose browning studies in human and rodent studies.

Model	Design	Factors Measured	Findings
Human	Tested 17 healthy subjects in 15–16 °C with increasing time to 6 h/day for 10 days with ^18^F-FDG-PET & abdominal subcutaneous fat biopsy [94]	Glucose uptake in BAT & WAT, energy expenditure, RMR, and NST.	Both females & males in cold exposure:
			NST ↑, Detectable BAT volume ↑.
			BAT activity ↑↑, Energy expenditure ↑.
Human	Single-blinded, placebo-controlled clinical trial. Exposed to 17 °C for 2 h/day for 6 weeks. Subjects with undetectable BAT were placed in cold (*n* = 12) and control (*n* = 10) groups. Capsinoid capsules with 9 or 0 mg/day given for 6 weeks. [136]	Body fat content; metabolic activity; EE; ^18^F-FDG-PET utilized.	After cold exposure: BAT activity ↑↑ in BAT + than in BAT− groups.
			Beige adipocytes recruited by BAT via cold & capsinoid treatment.
			Capsinoids → ↑↑ BAT thermogenesis & recruited BAT.
Human	A study between BAT & adiposity in 162 healthy volunteers (103 males & 59 females). ^18^F-FDG-PET/CT scan after 2 h at 19 °C. [42]	BMI, serum leptin, areas of visceral & subcutaneous fat.	41% of subjects were found with cold-activated BAT.
			Detectable BAT ↓ with ↑ age, with BAT found in 50% of 20-year-olds and >10% in 50-year-olds.
Human	Clinical trial with 15 subjects underwent cold exposure for 120 min at 14 °C. Treated with 200 mg/os mirabegron or placebo in a double blinded trial. [106]	Detected & quantified mirabegron via Agilent 6460 LC-MS/MS triple quadrupole mass spectrometer.	Cold exposure → ↑ Resting metabolic rate.
			Mirabegron → ↑ BAT glucose uptake.
			No correlation between drug- & cold-stimulation to measure BAT mass or activity.
Human	rospective study analyzed 5907 patients with ^18^F-FDG PET/CT scanning, but stringent standards → analysis of only 100 patients. [179]	Focal FDG uptake, blood glucose, liver fat content, lipid panel (Total, HDL, and LDL cholesterol; ALT; AST; triglycerides).	8 men and 17 women → ↑ brown fat uptake [ABAT (+) group].
			Total & LDL cholesterol ↓ in ABAT (+) vs. ABAT (−).
			Prevalence of NAFLD ↑ in ABAT (−) vs. ABAT (+).
			ABAT → ↓ serum transaminases (ALT & AST).
Human	10 lean & 14 overweight men on cold exposure for 2 h. ^18^F-FDG-PET scan for BAT activity. [7]	BAT activity & volume, skin & core temperature, BMI	BAT activity ↓ in obese subjects vs. lean subjects.
			BAT volume ↓ in obese subjects vs. lean subjects.
Human	6 non-acclimated men placed in a 10 °C environment for 2 h daily for 4 weeks. Subjected to electromyography and PET with [^11^C] acetate & [^18^F] FDG. [180]	Shivering intensity; BAT metabolism; fractional glucose uptake; insulin; triglycerides; T_3_; T_4_; ACTH; leptin levels	1.9-fold ↑ in thermogenic rate in cold exposure.
			Total BAT volume and activity ↑ by 45% after 4 weeks.
			Fractional glucose uptake ↑ in supraclavicular BAT.
Mouse	C57Bl/6 mice with FGF21-KO phenotype subjected → 5 °C for 72-h period. Extracted brown fat pads and subjected for RNA isolation, immunohistochemistry, and western blot analysis. [114]	Temperature, confluence of BAT in culture, PGC-1a mRNA expression.	Single-dose recombinant FGF21 → ↑ browning of WAT.
			Cold exposure & *β*3-AR stimulation ⇉ ↑ FGF21 in BAT & thermogenically competent WAT depots.
			FGF21 absence → impaired adaptive thermogenesis.
			PGC1-α required for FGF21’s function.
Mouse	Treated hESCs & hiPSCs to induce embryoid bodies and adipogenic differentiation for 21 days. Subcutaneously transplanted adipocytes in Rag2^−/−^; Il2yc mice. Infused FGF21 or saline pumps transplanted to the interscapular region. [181]	Lipolysis activity; adiponectin & leptin expression; glucose uptake; oxygen consumption	Converted hPSCs → MPCs → BAT & WAT
			Doxycycline treatment → ↑ PPARG2 → ↑↑ WAT phenotype. PPARG2-CEBPB-PRDM16 axis programmed BAT. Transplantation of hPSC-derived adipocytes → mature functionality.
Mouse	6–8 week-old mice fed with HFD for 8 weeks prior to BAT transplantation; no cold exposure. [182]	Body weight, basal glucose levels, and insulin tolerance.	BAT transplantation ⊣ HFD-induced obesity; ↑ Glucose tolerance; ↓ Insulin resistance. BAT removal → ↓ diet-induced obesity & insulin resistance.
Mouse	Study with strain-, sex-, and age-matched donor mice, whose BAT transplanted to other mice. Recipient mice fed on HFD, post transplantation for 20 weeks; cold exposure utilized. [15]	Body weight, total body fat (%), body temperature, O_2_ consumption, mRNA	BAT transplantation → ↓ HFD-induced weight gain;
			↓ Total fat; ↑ core body temperature; ↑ mRNA of FA oxidation-related genes; ↓ HFD-induced insulin resistance; ↓ HFD-induced hepatic steatosis & obesity.
Mouse	12-week-old male C57BL/6 mice as recipient mice fed standard food and were transplanted into the visceral cavity with 0.1 or 0.4 g BAT, or 0.1 g WAT from epididymal fat pad. [16]	Glucose concentration & uptake; food & water intake; CO_2_ & heat production; plasma lipids, hormones & proteins.	BAT transplantation → ↑ glucose tolerance & insulin sensitivity; ↑ BAT mass → ↑ metabolic homeostasis.
			BAT transplantation → more favorable circulating lipid and hormonal profile; ↑↑ circulating norepinephrine concentration & 5-fold increase in serum FGF21 levels.
Mouse	TZDs (Lobe, Rosi, and Pio) tested on cold acclimated 10-week-old C57BLKS/J-Leprdb/Leprdb male mice for 4 weeks. Raw264.7 macrophages & 3T3-cells treated with TZDs. [183]	Blood glucose; Glucose uptake; body weight; serum triglycerides, cholesterol, and FFA.	TZDs promoted adipocyte differentiation.
			Lobe stimulation >> two TZDs (Rosi & Pio) →↑↑ effect.
			Lobe → ↑ beige adipocyte formation & ↓ proinflammatory signals in leukocytes & adipocytes.
Mouse	After acclimation, 4- to 6-week-old C57BL/6J male mice put on high a fat diet for 8 weeks. Post-8 weeks, intraperitoneal injection of 8.5 ug/kg of recombinant follistatin (once/day for one week). [184]	Rectal temperature, mice activity; O_2_ consumption; CO_2_ production; glucose tolerance.	↓ HFD-induced obesity; ↑ insulin sensitivity.
			↑ subcutaneous fat browning via AMPK-PGC1a-irisin signaling pathway.
			↑ metabolism via insulin pathway in beige adipocytes.
Mouse	Male and female heterozygous mice used for mouse embryonic fibroblasts. C57BL/6 mice exposed to cold environment for 8 h. BAT harvested and compared to thermoneutral mice. 0.5 ug/uL of FST on brown preadipocytes for BAT differentiation. [124]	Genotyped embryos, gene and protein expressions of browning & metabolism-related genes; Oxygen consumption.	Follistatin → ↑ cellular respiration.
			Follistatin secreted by AT in a paracrine manner.
			Follistatin → ↑ brown adipocyte and thermogenic gene expression in differentiated brown adipocytes and MEF cultures from both WT and FST KO mice.
